# Cross-cultural adaptation and psychometric testing of the Yoruba lequesne algofunctional index of knee osteoarthritis among patients with knee osteoarthritis

**DOI:** 10.1186/s12891-023-07032-2

**Published:** 2023-11-18

**Authors:** Aderonke O. Akinpelu, Oluwatosin J. Omosanya, Adesola C. Odole, Babatunde O.A. Adegoke, Olufemi O. Oyewole

**Affiliations:** 1 Physiotherapy Department, University of Ibadan, Ibadan, Nigeria; 2 Physiotherapy Department, Olabisi Onabanjo University Teaching Hospital, Sagamu, Nigeria; 3 College of Health Sciences, University of KwaZulu-Natal, Private Bag X54001, Durban, South Africa

**Keywords:** Lequesne Algofunctional Index, Knee osteoarthritis, Validation, Cultural adaptation

## Abstract

**Background:**

The Lequesne Algofunctional Index of Knee Osteoarthritis (LAIKOA) is a widely used knee osteoarthritis (KOA) outcome measure and is recommended by many international authorities. It has been cross-culturally adapted to many languages, excluding indigenous Nigerian languages. The aim of this study was to cross-culturally adapt and validate the LAIKOA into Yoruba language.

**Methods:**

This was a validation study. Yoruba LAIKOA was translated and culturally adapted from English version following Beaton’s guidelines (including cognitive debriefing). The Yoruba LAIKOA was psychometrically tested for test-retest reliability, standard error of measurements (SEM), smallest detectable change (SDC), internal consistency, and construct validity among 108 Yoruba-speaking patients with KOA recruited from selected hospitals in Ibadan, Nigeria. Participants completed the Yoruba and English versions of LAIKOA, and the Yoruba version of Ibadan Knee/Hip Osteoarthritis Outcome Measure (IKHOAM).

**Results:**

The mean age of participants was 63.60 ± 11.77 years. Acceptable internal consistency was observed for the global index and function domain (α = 0.63–0.82) and good test-retest for items and domains (ICC = 0.81–0.995). Item-to-scale correlation was significant (r = 0.28–0.69). Its three domains demonstrated structural validity when subjected to confirmatory factor analysis (CFI = 0.99, TLI = 0.99, RMSEA = 0.02). Construct validity was supported by the correlation between Yoruba LAIKOA and IKHOAM (r = -0.39, p = 0.011). The overall scores and domain scores of the Yoruba and English versions of LAIKOA did not differ significantly. The Yoruba LAIKOA has no floor or ceiling effects.

**Conclusion:**

The Yoruba LAIKOA is reliable and valid, and it is recommended for use in clinical settings in southwestern Nigeria and other Yoruba-speaking populations.

## Background

Osteoarthritis (OA) is one of the most common chronic joint disorders worldwide [1]. The prevalence estimates for symptomatic osteoarthritis worldwide as of 2019 were 5.32% among men and 7.28% among women [2]. It is the leading cause of disability affecting 60–70% of the population older than 60 years. OA results in pain and physical disability, it has a significant impact on the health-related quality of life (HRQoL) of the patient [3]. The number of people affected with symptomatic knee OA was projected to increase because of the aging population and obesity epidemic [2]. Two community-based surveys in Southwest (age ≥ 40 years) and Northeast (age ≥ 30 years) Nigeria reported the prevalence of symptomatic knee OA to be 19.6% and 16.3% respectively. The two studies also reported a slight female bias of 1.2:1, and increasing age and body adiposity as predisposing factors to knee osteoarthritis [4, 5]. Adequate health care delivery for patients with knee osteoarthritis requires outcome measures for assessment of end result of patient care and its effect on the patient and the society [6].

Outcome measurement is the use of a tool to assess a patient’s current status [7]. Outcome measures may provide a score, an interpretation of results, and at times a risk categorization of the patient. Prior to providing any intervention, an outcome measure provides baseline data, which may help determine the course of treatment intervention. Once treatment has commenced, the same tool may be used in serial assessments to determine whether the patient has demonstrated a change in the symptoms and quality of life as a whole [8]. Credible and reliable justification for treatment on an individual patient level can be provided by efficient outcome measures. Measurement of outcomes in OA management refers to the assessment of different areas related to the degenerative changes in a joint and also the effect of these changes on the life of the patient [9]. Dimensions deemed to be important to patients included pain, function, quality of life, and activity level [10]. Outcome measurement, being a fundamental component of Evidence-Based Practice provides the necessary information clinicians require to make decisions in patient management [11]. The management of KOA as recommended by the American College of Rheumatology can be pharmacological, physical, psychosocial, and mind-body approaches [12].

The non-pharmacologic intervention in the management of knee OA includes rehabilitation-based weight reduction and physiotherapy such as strength training, aquatic exercise, Tai chi, Aerobic exercise, hydrotherapy, and Yoga [13]. Physiotherapy using various modalities and therapeutic exercises has been recommended for management of OA by the American College of Rheumatology (ACR) and the European League Against Rheumatism (EULAR) [14]. These interventions are aimed at relieving the symptoms of KOA, therefore assessment of various measures to monitor relief of symptoms and progress made on the management of KOA is important for effective management.

Many standardized OA outcome measures have been developed. These include the Arthritis Impact Measurement Scales (AIMS) [15], the Knee Osteoarthritis Outcome Score (KOOS) [16], the Hip disability and Osteoarthritis Outcome Score (HOOS) [17], the Western Ontario and McMaster University (WOMAC) Osteoarthritis Index [18], the Short Form 36 (SF 36), Arthritis Specific (ASHI) [19], the Ibadan Knee/Hip Osteoarthritis Outcome Measure (IKHOAM) [20], and the Lequesne Algofunctional Index for the severity of KOA [21].

The Lequesne Algofunctional Index of Knee Osteoarthritis (LAIKOA) was initially referred to as the Index for the severity of knee OA and it was developed originally in French language in the 1970s. The English version of the index was made available in 1987. In 1997 it was revised and renamed the Lequesne Algofunctional Index of knee osteoarthritis [21]. It is a patient report scale developed to assess patients’ pain and functional status [22]. It consists of 11 items in three domains which are pain or discomfort (5 items), maximum walking distance (2 items), and ADL or function (4 items) [21]. Although LAIKOA is widely used to determine the severity of knee osteoarthritis (KOA) in osteoarthritis clinical trials, it has also been used to assess the health-related quality of life of patients with KOA [23]. It is endorsed and recommended by international groups and authorities including Outcome Measures in Rheumatology (OMERACT), Osteoarthritis Research Society International (OARSI), and the United States Food and Drug Administration [23]. It has been shown that all items in the Lequesne Index are important for patients with OA in many cultures [24]. This index has been cross-culturally adapted to Singapore English, Chinese, Persian, Korean, German, and Bengali languages [22, 23, 25–27]. However, to the limit of our knowledge, LAIKOA is not available in any of the major indigenous Nigerian languages. The purpose of this study was to cross-culturally adapt the LAIKOA into the Yoruba language, the language of Southwest Nigeria, and to test some of its psychometric properties among Yoruba Patients with knee OA.

## Methods

This study was a validation study comprising translation and cultural adaptation and psychometric testing of the Yoruba version of LAIKOA. Participants for the psychometric testing were patients with KOA that have been diagnosed through clinical and physical tests, purposively recruited from the Surgical and Physiotherapy outpatient clinics, the Geriatrics rheumatology and Physiotherapy clinics of the Chief Tony Anenih Geriatric Centre, University College Hospital (UCH), Ibadan; and the Physiotherapy outpatient clinics of Adeoye Maternity, Yemetu, Ibadan. Participants were included if they were well oriented in time and space, could speak and understand either or both Yoruba and English and if they had symptoms and radiological evidence of KOA. Patients with knee OA and co-morbidities that could concurrently affect their health-related quality of life were excluded.

### Sample size

The sample size for psychometric testing of the Yoruba adapted LAIKOA was 108, as recommended by the consensus-based standards for the selection of health status measurement instruments [28]. However only 51 of the 108 patients with KOA could participate in the assessment of test-retest reliability of the scale. A sample of 50 is adjudged adequate for assessing test-retest reliability [28, 29].

### Instruments

Lequesne Algofunctional Index of knee OA: This English version of Lequesne Algofunctional Index of knee OA is a patient-reported (sometimes interviewer) outcome measure. This was cross-culturally adapted into Yoruba. The scale comprises 11 items in 3 domains, and each item is scored on a Likert scale of 0–2, except the maximum distance walked item, which is scored on a Likert scale of 1–6. The minimum score is zero (no functional limitation or pain) and maximum score is 24 (worse functional limitation or pain) [21].

Yoruba IKHOAM: This outcome measure which has already been validated in Yoruba language is a 3-domain/part, 33- item instrument [30]. The domains are the Activity limitations domain (part 1), Participation restrictions domain (part 2), and Physical performance tests domain (part 3). Parts 1 and 2 are patient-reported, which might be completed by the patient (self) or through an interview, while part 3 is clinician-measured. However, for the purpose of this study, only the activity limitation domain was completed by the participants. The activity limitation domain of IKHOAM assesses the degree of difficulty and the nature of assistance required to carry out each of its 25 items, and each item is scored on a Likert scale of 0–4, with minimum and maximum scores of 0 and 200 (scores 0 -100 for each of degree of difficulty and the nature of assistance required). The higher the score the worse the activity limitation.

### Procedure

The study was conducted in two stages as follows: Cross-cultural adaptation of the Lequesne Algofunctional Index of knee OA into Yoruba Language and Psychometric testing (Reliability and Validity) of the Yoruba version of Lequesne Algofunctional Index of knee OA.

Prior to the commencement of this study, an effort was made to contact the developer of the original version of the Lequesne Algofunctional Index of knee OA, Prof. M.G. Lequesne through email but to no avail. However, one of his collaborators (a co-author) Professor Mazieres Bernard was contacted via email to obtain his permission but his response was that because LAIKOA was already available in the public domain, seeking authors’ permission was not necessary.

The research protocol was approved by the University of Ibadan/ University College Hospital ethics committee (UI/UCH ethics committee assigned number: UI/EC/19/0425). Permission to conduct the study was obtained from the authorities of the clinics where the study was conducted. Informed consent was also obtained from each participant.

### Cross-cultural adaptation of the Lequesne Algofunctional Index of knee OA into the Yoruba language

The guidelines of the American Association of Orthopaedic Surgeons by Beaton et al. [31] were followed. The English version of the Lequesne Algofunctional Index of knee OA was translated into the Yoruba language by two independent bilingual translators who are proficient in English and Yoruba Languages and whose mother tongue is Yoruba. The first translator was provided with information and the concept involved in the Lequesne Algofunctional Index of knee OA, while the second translator was not given similar information. They produced two forward translations (T1 and T2). The two translators met to discuss and correct inconsistencies and differences in the two forward translations to produce a consensus translation (T12). The consensus Yoruba translation was back translated to English by two independent translators who were totally blinded to the process of forward translation. Two back translations BT1 and BT2 were produced.

An expert committee, comprising of the two forward translators, one of the backward translators, four musculoskeletal physiotherapists who are conversant with outcome measurement research was set up for the purpose of this study. One of the musculoskeletal physiotherapists served as the secretary to the committee. Each member of the committee was provided with copies of the source English version of the scale, forward translations (T1 and T2), consensus translation (T12), and back translations (BT1 and BT2). The committee examined the English version of the LAIKOA and the BT1 and BT2 along with T1, T2, and T12 for semantic, idiomatic, experiential and conceptual equivalences. The title, instructions, response scale and items were all examined. Consensus was reached each time an inconsistency between the English version and the backward translations was form. At the end of the meeting, the pre-final Yoruba version of LAIKOA was produced.

The pre-final Yoruba version of the Lequesne Algofunctional Index of knee OA was pre-tested on 30 Yoruba-speaking patients with knee OA. The LAIKOA (pre-final) was administered through interviews. The participants were also interviewed for cognitive debriefing interview on the relevance, clarity, and comprehension of instructions, items, and response options. Their comments and answers were considered in a second expert panel meeting and necessary modifications were made to the pre-final version to produce the final Yoruba version of the scale.

### Psychometric testing of the Yoruba version of lequesne algofunctional index of knee OA

The Yoruba version of the LAIKOA was investigated for, internal consistency, standard error of measurement (SEM), smallest detectable change (SDC), construct validity, and test-retest reliability. Each participant completed the English LAIKOA, the Yoruba LAIKOA and Yoruba version of IKHOAM [32]. In order to minimise bias in response, the order in which the three scales (the English LAIKOA, Yoruba adapted LAIKOA and the IKHOAM) were administered was varied. Each participant completed the third scale one hour after completing the other two scales. The Yoruba version of the Lequesne Algofunctional Index of knee OA was administered on a second occasion at three-day interval to assess the test-retest reliability of the scale (n = 51).

### Data analysis

Data were subjected to a normal distribution test and IKHOAM deviated from normality while LAIKOA did not. The missing data were replaced with series mean using replacing missing data of SPSS software. Socio-demographic variables and clinical characteristics of participants were summarized using mean and standard deviation, frequencies, and percentages. Paired t-test was used to test for differences between scores obtained on English and Yoruba versions of Lequesne Algofunctional Index of knee OA. Pearson correlation was used to estimate agreement between English and Yoruba versions while Spearman correlation was used to assess construct validity between Yoruba versions of LAIKOA and IKHOAM. Intraclass correlation Coefficient (single measures, two-way mixed effects, absolute agreement) between participants’ scores on two occasions was calculated to determine the test-retest reliability of the Yoruba version of Lequesne Algofunctional Index of knee OA. Standard error of measurement (SEM) and smallest detectable change (SDC) were also documented using the formula: SD$\sqrt{(1}-ICC)$ and 1.96$\sqrt{2}*SEM$, respectively (SD = average standard deviation of test and retest). Cronbach’s alpha values between each domain and the overall score were calculated to determine the internal consistency of the Yoruba version of the Lequesne Algofunctional Index of knee OA. Cronbach’s alpha and Intra class correlation Coefficient value of 0.7 is acceptable as a reliable instrument [29]. Confirmatory factor analysis and structural equation modeling was performed to examine the construct validity of the Yoruba version of LAIKOA using a maximum likelihood estimator. We examined the fit for the 1-factor and 3-factor models. Comparative fit index (CFI), Tucker-Lewis index (TLI), and root mean square error of approximation (RMSEA) were parameters used to assess model fit. The values of CFI and TLI ≥ 0.95 and RMSEA ≤ 0.05 are considered as good. The values of CFI and TLI ≥ 0.90 and RMSEA ≤ 0.08 are considered as acceptable [33]. Floor and ceiling effects were also calculated. If more than 15% of respondents achieved the lowest or highest possible score, the floor or ceiling effects are present [29]. The level of significance was set at 0.05. IBM SPSS Amos 28 software was used to analyze confirmatory factor analysis and structural equation modeling while IBM SPSS Statistics 28 was used for other analyses.

## Results

### Cross cultural adaptation of the lequesne algofunctional index of knee osteoarthritis into the Yoruba language

The expert committee observed that most of the items on the Yoruba translation of the Lequesne Algofunctional Index of knee osteoarthritis captured the concept of interest of the English version of the scale. However, five expressions in the Yoruba translation were found to be inconsistent with the English version. The expert panel committee corrected these inconsistencies and a pre-final version of the Yoruba LAIKOA was produced.

A total of 30 Yoruba patients with knee OA (20 females and 10 males) were involved in the pretesting procedure of the Yoruba LAIKOA with a mean age of 60.5 ± 10.2 years. All the respondents reported clarity of language and ease of understanding of the title, all items, and response options in the pre-final Yoruba LAIKOA during the cognitive debriefing interview.They understood the items in the pre-final version of Yoruba LAIKOA and the consensus at the second experts’ panel meeting was that no further adjustments should be made to the pre-final version.

### Psychometric testing of Yoruba LAIKOA

#### Demographic and clinical characteristics of participants

**Fig. 1:**
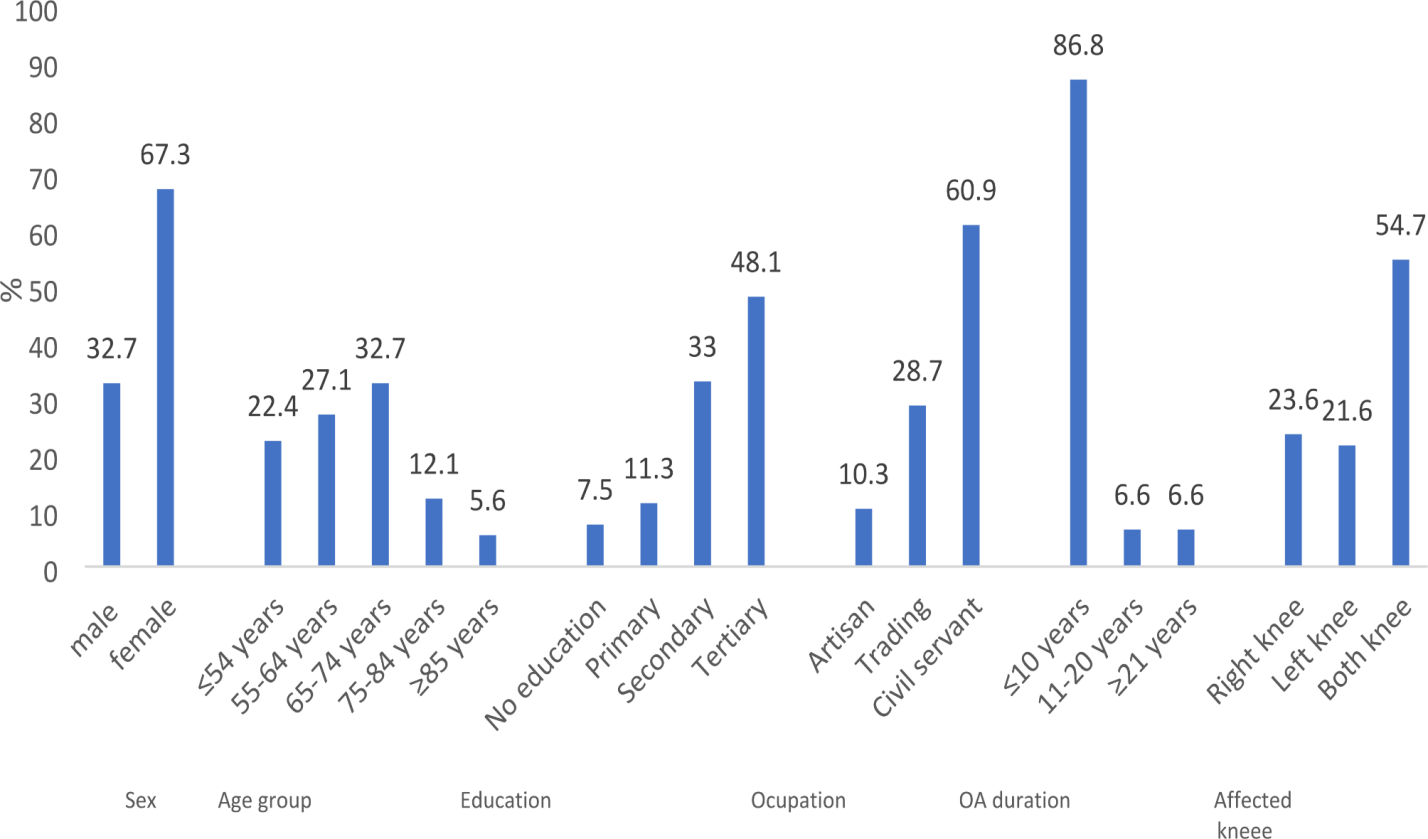
Characteristics of the participants

In the second phase of the study, 108 participants participated in the psychometric evaluation of the Yoruba LAIKOA. About 67% of the respondents were female; 27.1% were aged 55–64 years and 32.7% were aged 65–74 years. Their mean age was 63.60 ± 11.77 years and the median duration of knee OA was 4 years. About 61% of the participants were civil servants, 28.7% were traders and 10.3% were artisans. Most (86.8%) participants have had knee osteoarthritis for 10 years or less. More than half (54.7%) of participants had affectation of both knees. 48% of participants had tertiary education and 11.3% had primary education as their highest level of education (Fig. 1). The descriptive summary of Yoruba LAIKOA is shown in Table 1. The mean scores of items on Yoruba LAIKOA range from 0.41 to 2.53. Item 6 had the highest score (2.53) while item 7 had the lowest (0.41).

**Table 1:** Descriptive summary of items and domains of Yoruba LAIKOA

Item/domain	Mean (SD)	Median (IQR)	Min – Max	Skewness	Kurtosis
Q1	1.00 (0.71)	1.00 (0.25–1.75)	0.00–2.00	0.00	-0.99
Q2	0.75 (0.60)	1.00 (0.00–1.00)	0.00–2.00	0.15	-0.49
Q3	0.77 (0.42)	1.00 (1.00–1.00)	0.00 − 1.00	-1.29	-0.34
Q4	1.15 (0.78)	1.00 (1.00–1.00)	0.00–3.00	0.68	0.46
Q5	0.72 (0.45)	1.00 (0.00–1.00)	0.00 − 1.00	-0.98	-1.04
Q6	2.53 (1.99)	2.00 (1.00–4.00)	0.00–6.00	0.54	-0.88
Q7	0.41 (0.61)	0.00 (0.00–1.00)	0.00–2.00	1.22	0.47
Q8	0.70 (0.56)	0.50 (0.50-1.00)	0.00–2.00	0.56	-0.22
Q9	0.59 (0.54)	0.50 (0.00–1.00)	0.00–2.00	0.91	0.32
Q10	0.95 (0.62)	1.00 (0.50–1.50)	0.00–2.00	0.29	-1,07
Q11	0.78 (0.57)	0.50 (0.50 -1.00)	0.00–2.00	0.29	-1.07
Pain	4.38 (1.72)	5.00 (3.00–6.00)	0.00–8.00	-0.33	-0.19
Distance	2.94 (2.21)	2.00 (1.00–4.00)	0.00–8.00	0.61	-0.59
Function	3.02 (1.85)	3.00 (1.50-4.00)	0.00–8.00	0.76	0.11
Total	10.35 (4.20)	10.50 (8.00–12.00)	0.00–23.00	0.24	0.48

### Psychometric properties

Table 2 shows the psychometric properties of the Yoruba LAIKOA. It demonstrates moderate internal consistency with an alpha value of 0.63. However, the function domain of Yoruba LAIKOA shows good internal consistency (α = 0.82). If an item was deleted, the internal consistency is within the acceptable range (α = 0.57 to 0.72). The overall construct validity for the Yoruba LAIKOA was fair (r=-0.39) and fair across the domains with exception of the pain domain.

**Table 2:** Psychometric properties of Yoruba version of LAIKOA

Item/domain	Yoruba LAIKOA				Yoruba with English LAIKOA	Construct validity	
	Corrected Item-Total Correlation	Cronbach’s Alpha if Item Deleted	Cronbach’s Alpha	ICC (95% CI)	ICC (95% CI)	r	p-value
Q1	0.266	0.614		0.86 (0.77–0.92)	0.61 (0.48–0.72)		
Q2	0.224	0.621		0.97 (0.95–0.98)	0.71 (0.60–0.79)		
Q3	0.328	0.613		0.81 (0.69–0.89)	0.46 (0.30–0.60)		
Q4	0.097	0.644		0.96 (0.92–0.98)	0.64 (0.51–0.74)		
Q5	0.309	0.614		0.81 (0.69–0.89)	0.56 (0.42–0.68)		
Q6	0.286	0.719		0.995 (0.99–0.997)	0.76 (0.67–0.83)		
Q7	0.333	0.605		0.98 (0.96–0.99)	0.75 (0.66–0.82)		
Q8	0.540	0.578		0.88 (0.80–0.93)	0.66 (0.53–0.75)		
Q9	0.565	0.576		0.94 (0.90–0.96)	0.64 (0.51–0.74)		
Q10	0.491	0.580		0.92 (0.87–0.95)	0.67 (0.56–0.76)		
Q11	0.579	0.571		0.97 (0.95–0.98)	0.77 (0.67–0.83)		
Pain			0.467	0.87 (0.77–0.93)	0.66 (0.53–0.75)	0.102	0.522
Distance			0.235	0.995(0.99–0.997)	0.81 (0.73–0.87)	-0.455	0.002
Function			0.821	0.95 (0.92–0.97)	0.75 (0.66–0.83)	-0.390	0.011
Total			0.632	0.96 (0.93–0.98)	0.74 (0.64–0.81)	-0.389	0.011

LAIKOA: Lequesne Algofunctional Index of knee osteoarthritis

**Fig. 2:**
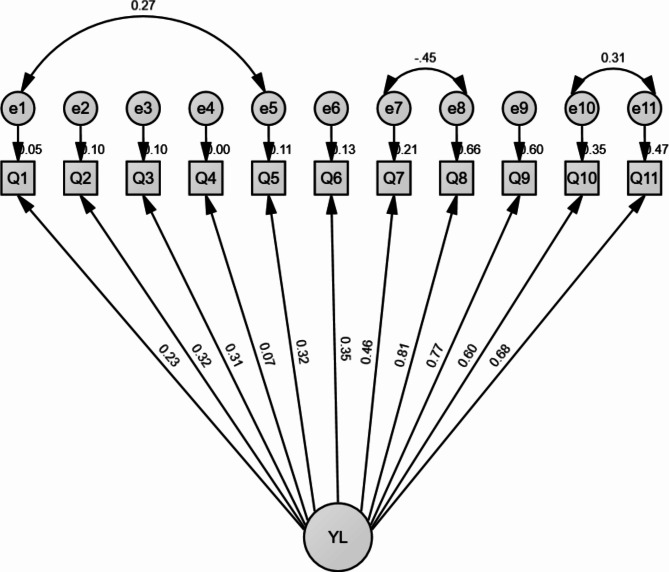
1-Factor structural equation modeling for Yoruba LAIKOA

**Fig. 3:**
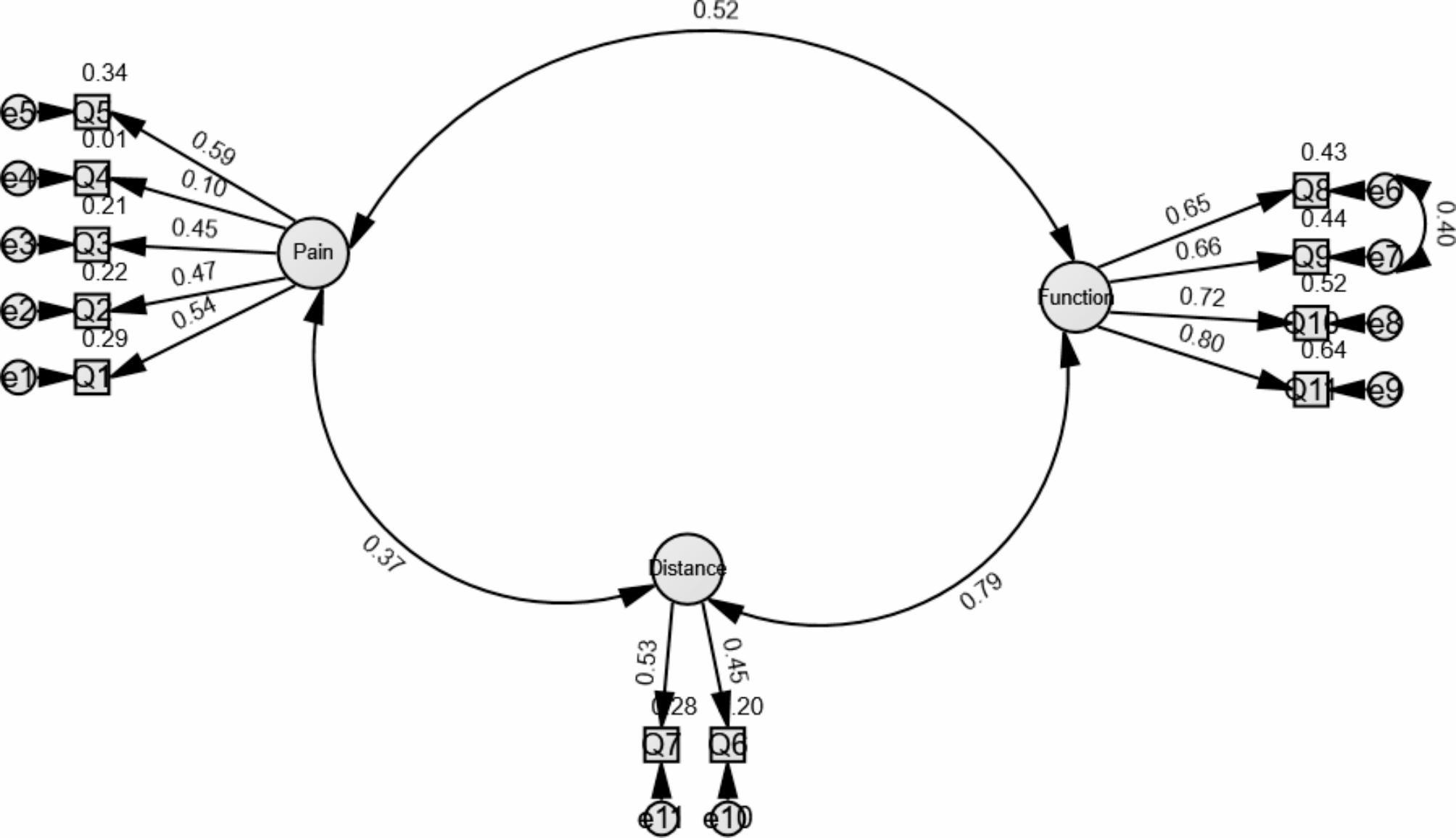
3-Factor structural equation modeling for Yoruba LAIKOA

Confirmatory factor analysis of Yoruba LAIKOA is shown in Table 3; Figs. 2 and 3. The three domains of the Yoruba LAIKOA demonstrated structural validity (Fig. 3). The 3-factor model returned a good fit after one error modification [e6 with e7 (0.40), CFI = 0.99, TLI = 0.99, RMSEA = 0.02] (Table 3; Fig. 3). The factor loadings were good for the items save item 4 in the pain domain. However, the Yoruba LAIKOA can be used as a single tool as the 1-factor model returned a good fit after modification [maximum of three errors, e1 with e5 (0.27), e7 with e8 (-0.45) and e10 with e11(0.31), CFI = 0.97, TLI = 0.96, RMSEA = 0.04] (Fig. 2; Table 3). The composite reliability of both models was good, 0.828 and 0.748 for 3-factor and 1-factor, respectively.

**Table 3:** Confirmatory factor analysis for Yoruba LAIKOA

Item	1-factor confirmatory factor analysis of Yoruba LAIKOA			3-factor confirmatory factor analysis of Yoruba LAIKOA		
	Factor loading	R^2^	Composite reliability	Factor loading	R^2^	Composite reliability
Q1	0.228	0.052	0.788	0.537	0.288	0.882
Q2	0.322	0.103		0.474	0.224	
Q3	0.312	0.098		0.454	0.206	
Q4	0.069	0.005		0.105	0.011	
Q5	0.325	0.105		0.586	0.343	
Q6	0.354	0.125		0.446	0.199	
Q7	0.463	0.214		0.532	0.283	
Q8	0.811	0.658		0.653	0.426	
Q9	0.774	0.599		0.664	0.441	
Q10	0.595	0.354		0.721	0.519	
Q11	0.682	0.466		0.801	0.641	
Fit indices	χ^2^ = 47.93, P = 0.212, CFI = 0.97, TLI = 0.96, RMSEA = 0.04 (0.00-0.08)			χ^2^ = 42.10, P = 0.380, CFI = 0.99, TLI = 0.99, RMSEA = 0.02 (0.00-0.07)		

LAIKOA: Lequesne Algofunctional Index of knee osteoarthritis

The test and retest mean (SD) of Yoruba LAIKOA were 11.03 (4.62) and 10.62 (4.52) with a mean difference of 0.41(1.27). The test-retest reliability of Yoruba LAIKOA was good to excellent with ICC values ranging from 0.81 to 0.995. It was excellent for items 2, 4, 6–7, 9–11 and distance and function domains. The overall intraclass correlation coefficient between participants’ scores on the Yoruba version of LAIKOA on two occasions was significant (ICC = 0.96) (Table 2). The two measurements (test and retest) were significant from one another (p = 0.025). Thus, the Bland Altman plot cannot be plotted. The standard error of measurement was 0.937 and the smallest detectable change was 2.597. Only 0.9% of the participants obtained the lowest scores while no ceiling effect was observed.

The overall scores of the Yoruba version (10.35 ± 4.20) and English version (10.44 ± 3.79) of LAIKOA did not differ significantly (t=-0.315, p = 0.754). Each of the domain scores on the Yoruba version of LAIKOA also showed no significant difference when compared to the English version of LAIKOA. Comparison between each item’s scores of Yoruba and English LAIKOA showed no significant difference except for items 3 and 4 (Table 4).

**Table 4:** Comparison of items and domains of Yoruba and English versions of LAIKOA

Item/domain	Yoruba LAIKOA	English LAIKOA	t- statistics	p-value
Q1	1.00 ± 0.71	1.06 ± 0.67	-1.025	0.308
Q2	0.75 ± 0.60	0.78 ± 0.62	-0.624	0.534
Q3`	0.77 ± 0.42	0.63 ± 0.49	3.119	0.002
Q4	1.15 ± 0.78	0.76 ± 0.43	4.465	< 0.001
Q5	0.72 ± 0.45	0.76 ± 0.43	-1.054	0.294
Q6	2.53 ± 1.99	2.57 ± 1.83	-0.328	0.743
Q7	0.41 ± 0.61	0.34 ± 0.52	1.825	0.071
Q8	0.70 ± 0.56	0.72 ± 0.54	-0.528	0.598
Q9	0.59 ± 0.54	0.51 ± 0.50	1.967	0.052
Q10	0.95 ± 0.62	1.01 ± 0.62	-1.248	0.215
Q11	0.78 ± 0.57	0.76 ± 0.54	0.628	0.531
Pain	4.38 ± 1.72	4.52 ± 1.58	-1.055	0.294
Distance	2.94 ± 2.21	2.92 ± 2.08	0.217	0.829
Function	3.02 ± 1.85	3.00 ± 1.76	0.189	0.851
Total	10.35 ± 4.20	10.44 ± 3.79	-0.315	0.754

LAIKOA: Lequesne Algofunctional Index of knee osteoarthritis

Table 5 shows the correlation matrixes of Yoruba and English versions of LAIKOA and its items. The Yoruba and English LAIKOA were significantly correlated (r = 0.74). Its domains (r = 0.66–0.81) and items (r = 0.56–0.77) have a good correlation with each other. Each item of Yoruba LAIKOA was significantly correlated with total scores with correlation matrixes ranging between 0.28 and 0.69.

**Table 5:** correlation matrix of items and domains of Yoruba and English LAIKOA

	Pain domain	distance domain	Function domain	Total score	Q1	Q2	Q3	Q4	Q5	Q6	Q7	Q8	Q9	Q10	Q11
Pain domain															
	0.659^**^	-0.073	0.301^**^	0.364^**^	0.392^**^	0.467^**^	0.323^**^	0.399^**^	0.283^**^	-0.100	0.060	0.294^**^	0.221^*^	0.226^*^	0.233^*^
distance domain	0.125														
	-0.011	0.810^**^	0.305^**^	0.557^**^	0.000	-0.085	0.031	0.008	0.030	0.759^**^	0.465^**^	0.221^*^	0.375^**^	0.120	0.286^**^
Function domain	0.354^**^	0.380^**^													
	0.167	0.395^**^	0.753^**^	0.609^**^	0.123	0.154	0.142	0.004	0.098	0.340^**^	0.325^**^	0.564^**^	0.620^**^	0.573^**^	0.678^**^
Total score	0.633^**^	0.746^**^	0.787^**^												
	0.345^**^	0.597^**^	0.642^**^	0.740^**^	0.220^*^	0.219^*^	0.217^*^	0.172	0.180	0.532^**^	0.431^**^	0.505^**^	0.586^**^	0.427^**^	0.569^**^
Q1	0.710^**^	0.080	0.195^*^	0.420^**^											
	0.446^**^	-0.029	0.135	0.227^*^	0.610^**^	0.149	0.108	0.000	0.153	-0.014	0.051	0.061	0.079	0.137	0.145
Q2	0.557^**^	0.025	0.262^**^	0.357^**^	0.286^**^										
	0.310^**^	-0.170	0.196^*^	0.124	0.225^*^	0.710^**^	0.161	-0.063	0.273^**^	-0.107	0.038	0.116	0.149	0.082	0.110
Q3	0.558^**^	0.146	0.250^**^	0.416^**^	0.248^**^	0.175									
	0.340^**^	-0.011	0.205^*^	0.224^*^	0.116	-0.091	0.488^**^	0.221^*^	0.204^*^	-0.008	0.153	0.186	0.121	0.061	0.189
Q4	0.519^**^	0.048	0.095	0.280^**^	0.118	-0.060	0.189								
	0.321^**^	0.004	0.139	0.195^*^	0.090	-0.028	0.244^*^	0.650^**^	-0.032	0.045	-0.127	-0.012	-0.028	0.055	0.091
Q5	0.537^**^	0.099	0.298^**^	0.404^**^	0.322^**^	0.291^**^	0.242^*^	-0.039							
	0.341^**^	0.025	0.141	0.215^*^	0.181	0.142	0.030	-0.027	0.564^**^	0.011	0.079	0.068	0.012	0.051	0.103
Q6	0.104	0.963^**^	0.315^**^	0.690^**^	0.079	-0.005	0.092	0.075	0.063						
	-0.022	0.773^**^	0.269^**^	0.517^**^	-0.024	-0.185	-0.036	-0.013	-0.002	0.764^**^	0.355^**^	0.355^**^	0.401^**^	0.141	0.244^*^
Q7	0.115	0.489^**^	0.351^**^	0.460^**^	0.032	0.106	0.228^*^	− 0.070	0.155	0.237^*^					
	0.035	0.530^**^	0.278^**^	0.416^**^	-0.027	-0.015	0.078	0.058	0.097	0.314^**^	0.766^**^	0.244^*^	0.264^**^	0.229^*^	0.292^**^
Q8	0.330^**^	0.260^**^	0.811^**^	0.630^**^	0.190^*^	0.292^**^	0.198^*^	0.093	0.227^*^	0.246^*^	0.142				
	0.123	0.386^**^	0.611^**^	0.524^**^	0.087	0.253^**^	0.259^**^	0.117	0.085	0.218^*^	0.119	0.654^**^	0.416^**^	0.272^**^	0.478^**^
Q9	0.266^**^	0.346^**^	0.812^**^	0.650^**^	0.145	0.259^**^	0.216^*^	0.000	0.242^*^	0.292^**^	0.306^**^	0.659^**^			
	0.105	0.433^**^	0.558^**^	0.518^**^	0.100	0.174	0.131	0.071	0.136	0.351^**^	0.270^**^	0.543^**^	0.645^**^	0.364^**^	0.433^**^
Q10	0.284^**^	0.250^**^	0.795^**^	0.599^**^	0.117	0.205^*^	0.168	0.112	0.275^**^	0.180	0.322^**^	0.431^**^	0.508^**^		
	0.138	0.190^*^	0.568^**^	0.407^**^	0.103	0.105	0.092	0.166	0.129	0.076	0.217^*^	0.321^**^	0.328^**^	0.674^**^	0.497^**^
Q11	0.265^**^	0.375^**^	0.817^**^	0.667^**^	0.184	0.095	0.229^*^	0.095	0.217^*^	0.308^**^	0.357^**^	0.559^**^	0.487^**^	0.587^**^	
	0.710^**^	0.080	0.195^*^	0.420^**^	0.145	0.110	0.189	0.091	0.103	0.244^*^	0.292^**^	0.478^**^	0.433^**^	0.497^**^	0.765**

**. Correlation is significant at the 0.01 level (2-tailed)

*. Correlation is significant at the 0.05 level (2-tailed)

LAIKOA: Lequesne Algofunctional Index of knee osteoarthritis

The first line (row) of values reflects correlations derived from the items and domain of Yoruba version of LAIKOA and the second line represents correlations between English and Yoruba version of LAIKOA.

## Discussion

The process of cross-cultural adaptation of the Lequesne Algofunctional Index of knee osteoarthritis into the Yoruba language in this study was according to the guideline recommended by the American Association of Orthopedic Surgeons (AAOS) [31]. After much discussion about the challenge of getting a direct translation of the word ‘algofunctional’ which would be clumsy, the expert panel decided on a title that was agreed to have an excellent conceptual equivalence with the title of the English version. The back translations of the consensus Yoruba version of the LAIKOA were consistent with the English version of the LAIKOA for almost all the items. The function domain’s response options were also corrected to cut across the whole items in the domain just like the English version of the LAIKOA.

The cross-cultural adaptation process of LAIKOA into the Yoruba language confirms that all stages involved in the adaptation as proposed by Beaton et al. [31] are important for a successful adaptation of a scale into another language. Kulhawy-Wibe et al. [34] confirmed in their systematic review and appraisal of the cross-cultural validity of functional status assessment measures in rheumatoid arthritis that all these stages are important processes to enhance methodological rating as proposed by COSMIN [34].

The mean age (63.60 ± 11.77 years) of KOA patients in this study supports the fact that KOA is an age-related problem with its greatest risk factor as older age [35, 36]. The sex distribution of participants in this study showed a female preponderance which is in accord with findings from the previous studies [5, 37].

The construct validity of the Yoruba version of LAIKOA was assessed by testing for differences between the domain and overall scores obtained on the English version and the Yoruba version of the LAIKOA. The findings of no significant difference between the English version and the Yoruba version of the LAIKOA show that the Yoruba version of LAIKOA measures the same construct as the English version which has been validated [21]. There was also a significant negative correlation between the function domain of the Yoruba version of LAIKOA and the Activity limitation of the Yoruba version of IKHOAM, which implies that the measure of Activity limitation by Yoruba IKHOAM correlates with the measure of Activities of Daily Living by the Yoruba version of LAIKOA. This result is supported by the valid translation of LAIKOA into Korean, Bengali, Singapore English, Moroccan Arabic, Chinese, Turkish, and Portuguese languages [22, 23, 25, 26, 38–40]. However, these studies validated their translations using WOMAC [26, 39, 40], SF-36 [22, 23], SF-20 [27], EQ-5D [23], and VAS [22, 25, 27] which have been translated into their languages and validated in their culture.

The values of Cronbach’s alpha suggest that there was poor internal consistency in the distance domain and fair in the pain domain of the Yoruba LAIKOA. However, good internal consistency was observed in the function domain. This is in accordance with previous studies, Xie et al. [23] found a Cronbach’s alpha of 0.44 in the Pain domain of the Singapore Chinese version of LAIKOA which is similar to the findings of fair internal consistency in the Pain domain of the Yoruba version of LAIKOA. The same results were shown in the Singapore English (0.58) [23], Turkish (0.61) [38], and Persian (0.635) [27] versions of LAIKOA. A good internal consistency (0.821) was found in the function domain of the Yoruba version of LAIKOA, this is similar to the findings of the Turkish (0.71), Persian (0.761), Moroccan Arabic (0.90), Korean (0.85), Bengali (0.91), Chinese (0.77) and Singapore English (0.82) versions of the LAIKOA [22, 23, 25, 27, 39, 40]. These studies did not test for the internal consistency of the distance domain. However, the low level of internal consistency observed in the distance domain could be as a result of the varying grading schedules adopted in the other two domains compared to the function domain.

Confirmatory factor analysis and structural equation modeling returned 1-factor and 3-factor models. Although the scale was designed originally to be unidimensional and used as a single tool [21], the 1-factor model seems to confirm this as it demonstrated a good fit. The unidimensional of the scale was further buttressed by the value of global Cronbach alpha (α) and alpha (0.57–0.72) if the item was deleted as these values demonstrated good internal consistency of a single scale. Other studies have argued that the scale is not unidimensional [22, 23, 40–42]. These studies found two extracted factors though, the items could not be easily grouped into two meaningful clinical factors [22, 23, 40]. However, Faucher et al. [41] and Dawson et al. [42] reported two distinct clinical factors of pain and function. Our 3-factor model seems to corroborate that Yoruba LAIKOA has three domains structurally. The fit indices were good for the 3-factor model supporting the multidimensional of Yoruba LAIKOA.

The ICC between respondents’ overall scores on two occasions indicated that the Yoruba version of LAIKOA demonstrated excellent test-retest reliability (ICC = 0.96). This was higher than the ICC for the Korean version of LAIKOA (ICC = 0.87) [25], the German version (ICC = 0.88) [26], and the Persian version (ICC = 0.84) [27]. The report of excellent test-retest reliability (ICC = 0.97) of Bangali version of LAIKOA by Mahmood et al. [22] is similar to our finding of ICC = 0.96. Again, our data suggest the uncompromising reliability of the Yoruba version of LAIKOA as the floor or ceiling effects were not observed.

The SEM of the Yoruba version of LAIKOA of 0.94 points represents 3.92% of the possible range of overall scores (0–24) while SDC of 2.60 points represents 10.83% of the range of possible scores. This may imply that patients with an SDC score ≥ 2.60 have a 95% probability that a real change occurred, and that it is not due to measurement error. The probability that real change does not occur is less than 5% is small in day-to-day clinical experience; thus, a score of 2.60 could be a real change. This low measurement error implies that the Yoruba LAIKOA is a good measure of pain and function in the OA population in a clinical setting. This satisfied the COSMIN guideline that patient-reported outcomes in routine clinical practice must have minimal measurement error [28].

The present study has a few limitations. The small sample size in this study may reduce the power of the study. However, the COSMIN checklist judged the sample size adequate for construct validity. This study did not assess the responsiveness of the Yoruba version of LAIKOA. The study only provides a preliminary investigation of psychometric properties of the Yoruba version of LAIKOA.

## Conclusion

The Yoruba version Lequesne Algofunctional Index of knee osteoarthritis demonstrates a 3-factor model structurally and is a reliable and valid scale for the assessment of knee osteoarthritis in southwestern Nigeria and other Yoruba-speaking populations.

## Data Availability

The datasets generated during and/or analysed during the current study are available from the corresponding author on reasonable request.

## List of abbreviations

LAIKOA: Lequesne Algofunctional Index of Knee Osteoarthritis
KOA: Knee Osteoarthritis
IKHOAM: Ibadan Knee/Hip Osteoarthritis Outcome Measure
OA: Osteoarthritis
HRQoL: Health-Related Quality of Life
ACR: American College of Rheumatology
EULAR: European League Against Rheumatism
AIMS: Arthritis Impact Measurement Scales
KOOS: Knee Osteoarthritis Outcome Score
HOOS: Hip disability and Osteoarthritis Outcome Score
WOMAC: Western Ontario and McMaster University Osteoarthritis Index
SF-36: Short Form 36
ASHI: Arthritis Specific
OMERACT: Outcome Measures in Rheumatology
OARSI: Osteoarthritis Research Society International
UCH: University College Hospital
UI: University of Ibadan
T1: Forward Translations 1
T2: Forward Translations 2
T12: Consensus Forward Translations 12
BT1: Back Translations 1
BT2: Back Translations 2
CFI: Comparative Fit Index
TLI: Tucker-Lewis Index
RMSEA: Root Mean Square Error of Approximation
ICC: Intraclass Correlation Coefficient
AAOS: American Association of Orthopedic Surgeons
SEM: Standard Error of Measurement
SDC: Smallest Detectable Change
